# An integrated CSF-serum biomarker model for predicting clinical progression in Alzheimer’s disease

**DOI:** 10.3389/fnagi.2026.1728675

**Published:** 2026-01-27

**Authors:** Xichun Wang, Ye Tang, Qiwen Zhang, Baozhen Xiang, Silin Zeng, Qian Zhang, Mei Gu, Liangyu Zou

**Affiliations:** 1 Department of Neurology, Shenzhen People’s Hospital, The Second Clinical Medical College, Jinan University, Shenzhen, China; 2 Jinan University, Guangzhou, China; 3 Department of Neurology, Shenzhen People’s Hospital (The First Affiliated Hospital, Southern University of Science and Technology; The Second Clinical Medical College, Jinan University), Shenzhen, China

**Keywords:** albumin-to-globulin ratio, Alzheimer’s disease, nomogram, platelet-to-lymphocyte ratio, pTau181

## Abstract

**Background:**

The early and accurate identification of Alzheimer’s disease (AD) remains a significant clinical challenge. Integrating novel peripheral blood-based biomarkers with established cerebrospinal fluid (CSF) measures may offer a promising strategy to enhance diagnostic accuracy and risk stratification.

**Methods:**

This study enrolled 91 participants who underwent CSF and serum testing. The cohort was randomly divided into a training set (*n* = 63) and an internal testing set (*n* = 28). External validation was performed using matched data (*n* = 30) from the Alzheimer’s Disease Neuroimaging Initiative (ADNI) database (total *n* = 639). Data collected included demographics, Mini-Mental State Examination (MMSE) total scores, the Functional Activities Questionnaire (FAQ) total scores, CSF phosphorylated tau (pTau181) and amyloid-*β* (Aβ42) levels, and serum indices such as the albumin-to-globulin (A/G) ratio and platelet-to-lymphocyte ratio (PLR). Predictor selection was performed via univariate and multivariate logistic regression, and a nomogram was developed from the final model. Model performance was evaluated using the area under the receiver operating characteristic curve (AUC), calibration curves with mean absolute error (MAE), and decision curve analysis (DCA).

**Results:**

The final predictive model incorporated CSF pTau181, A/G ratio, and PLR (using a cut-off ≥113.22). It demonstrated robust discrimination, achieving an AUC of 0.92 in the training set, 0.86 in the testing set, and 0.83 upon external validation. Calibration was excellent (MAE = 0.039). In the testing set, sensitivity was 0.83 and specificity was 0.86. A higher A/G ratio was associated with a reduced risk of AD progression, whereas a higher PLR was associated with an increased risk.

**Conclusion:**

The combined CSF-peripheral blood biomarker model demonstrates robust discrimination and calibration for predicting AD progression. By linking central tau pathology with peripheral nutritional and inflammatory status, it may aid clinical risk stratification and guide management strategies focused on nutrition and inflammation. Further large-scale, prospective validation is warranted.

## 1 Introduction

Alzheimer’s disease (AD) is a progressive neurodegenerative disorder characterized by a gradual decline in cognitive function, frequently accompanied by neuropsychiatric symptoms in its later stages (McDade, 2022). Although the precise pathogenesis of AD remains incompletely understood (Tatulian, 2022), its biological definition has become increasingly well-established. Hallmark neuropathological changes can now be detected *in vivo* using cerebrospinal fluid (CSF), blood, or neuroimaging biomarkers, enabling a biological diagnosis of AD based on a positive biomarker profile, irrespective of the presence or severity of clinical symptoms (Jack et al., 2018; Jack et al., 2024).

In 2024, the National Institute on Aging-Alzheimer’s Association (NIA-AA) revised its diagnostic and staging criteria, emphasizing the biological foundation of AD diagnosis and introducing a six-stage numeric clinical staging system to more precisely characterize the severity of cognitive and functional impairments along the AD continuum. This updated framework redefines previously used concepts, such as a “prodrome of AD” or “at risk for AD, “into more clinically applicable categories: Clinical Stage 2, denoting mild detectable change with minimal functional impact, and Stage 3, representing mild cognitive impairment (MCI) with early functional limitations (Jack et al., 2024).

Accumulating evidence suggests that AD pathophysiology may commence decades before overt clinical symptoms appear (Guzman-Martinez et al., 2021). Therefore, early detection of individuals in Clinical Stages 1–2 (asymptomatic or minimally symptomatic) and Stage 3 (MCI) is critical for timely intervention (Petersen et al., 2014). With the recent approval and widespread implementation of disease-modifying therapies (DMTs), such as anti-Aβ monoclonal antibodies like lecanemab, accurately detecting these early-stage individuals and initiating DMTs promptly have become essential strategies for slowing disease progression and preserving cognitive function (Tahami Monfared et al., 2022; Cohen et al., 2023; Tahami Monfared et al., 2023). Recent studies further indicate that biomarker-based predictive models can aid in the early identification of high-risk individuals, supporting interventions targeting modifiable risk factors, and offering new prospects for primary prevention and early intervention in AD (Graff-Radford et al., 2021; van Oostveen and de Lange, 2021).

CSF biomarkers, including amyloid-beta (Aβ42), total tau (t-Tau), and phosphorylated tau (pTau181), are central to the biological diagnosis of AD (Jack et al., 2018; Jack et al., 2024). In particular, CSF and plasma pTau181 have emerged as highly specific indicators of AD-related tau pathology. Critically, elevated levels of pTau181, irrespective of the specific assay platform used, have been consistently and strongly associated with the presence and severity of AD across numerous independent cohorts and studies (Janelidze et al., 2020; Lv et al., 2023). However, the absolute value of CSF pTau181 in an individual patient does not exhibit a perfect linear relationship with current symptom severity, especially in cases with comorbidities such as vascular dementia, where clinical manifestations may be influenced by mixed pathologies. This complexity limits the utility of pTau181 alone for assessing AD severity. For these reasons, the 2024 NIA-AA criteria introduced three additional biomarker categories: inflammatory/immune mechanisms (I), vascular brain injury (V), and *α*-synuclein (S) (Jack et al., 2024). Among these, the “I” category includes biomarkers such as glial fibrillary acidic protein (GFAP), which reflects non-specific inflammatory processes involved in AD pathophysiology. Nevertheless, the relatively high cost of GFAP testing limits its accessibility, underscoring a pressing need to develop complementary, cost-effective, and non-invasive tools to support clinical staging and prognostic assessment (Benedet et al., 2021; Pereira et al., 2021; Jack et al., 2024).

In recent years, peripheral inflammatory markers have garnered increasing attention in AD research (Koyama et al., 2013). Accumulating evidence suggests that systemic inflammation, metabolic dysregulation, and nutritional deficiencies may influence both disease progression and clinical expression of AD and cognitive impairment (Koyama et al., 2016; Maeda et al., 2019; Zhou et al., 2021; Wang et al., 2024; Yang et al., 2024). Such readily obtainable serum biomarkers could thus serve as a practical and affordable adjunct to CSF analysis. We hypothesize that combining CSF pTau181 with serum biomarkers will yield a robust and clinically applicable model for distinguishing between early (Stages 1–2) and later (Stages 3–6) clinical stages of AD. Therefore, this study aims to develop and validate a novel combinatorial model to improve the accuracy of predicting clinical progression (Koyama et al., 2013; van der Lee et al., 2018).

Notably, nomograms have been widely used as precise instruments for predicting disease outcomes in AD and other disorders (Chen et al., 2025). These graphical tools, derived from regression models, estimate the probability of a clinical event based on multiple predictive variables and are increasingly regarded as a potential alternative or even a new standard compared to traditional staging systems (Balachandran et al., 2015; Lv et al., 2022). In this study, we aimed to develop and validate a nomogram to distinguish AD patients with objective cognitive impairment (Clinical Stages 3–6) from those without (Clinical Stage 1–2 AD).

## 2 Methods

### 2.1 Study design and participants

This study was conducted following the guidelines provided in the 2024 edition of “Developing Clinical Prediction Models: A Step-by-Step Guide” (Efthimiou et al., 2024), which outlines methodological standards for developing and validating diagnostic or prognostic prediction models. We retrospectively enrolled 103 patients who underwent lumbar puncture for cognitive assessment and CSF AD biomarker analysis at the Department of Neurology, Shenzhen People’s Hospital between 2019 and 2025. After excluding 12 patients due to incomplete data—specifically missing Mini-Mental State Examination (MMSE) scores or serum biomarker measurements—the final analytical cohort comprised 91 participants. Consistent with contemporary recommendations for feasible operationalization in clinical research (Selma-Gonzalez et al., 2025), the Mini-Mental State Examination (MMSE) was adopted as the principal criterion for cognitive staging. Functional status was concurrently assessed across all participants using the Functional Activities Questionnaire (FAQ), a useful method to discriminate and predict progression from clinical normal to MCI (Marshall et al., 2015). The resulting FAQ scores served to both characterize and provide concurrent validation that the MMSE-based stratification effectively captured the anticipated gradient in functional impairment—a dimension central to distinguishing these clinical stages.

**Figure 1:**
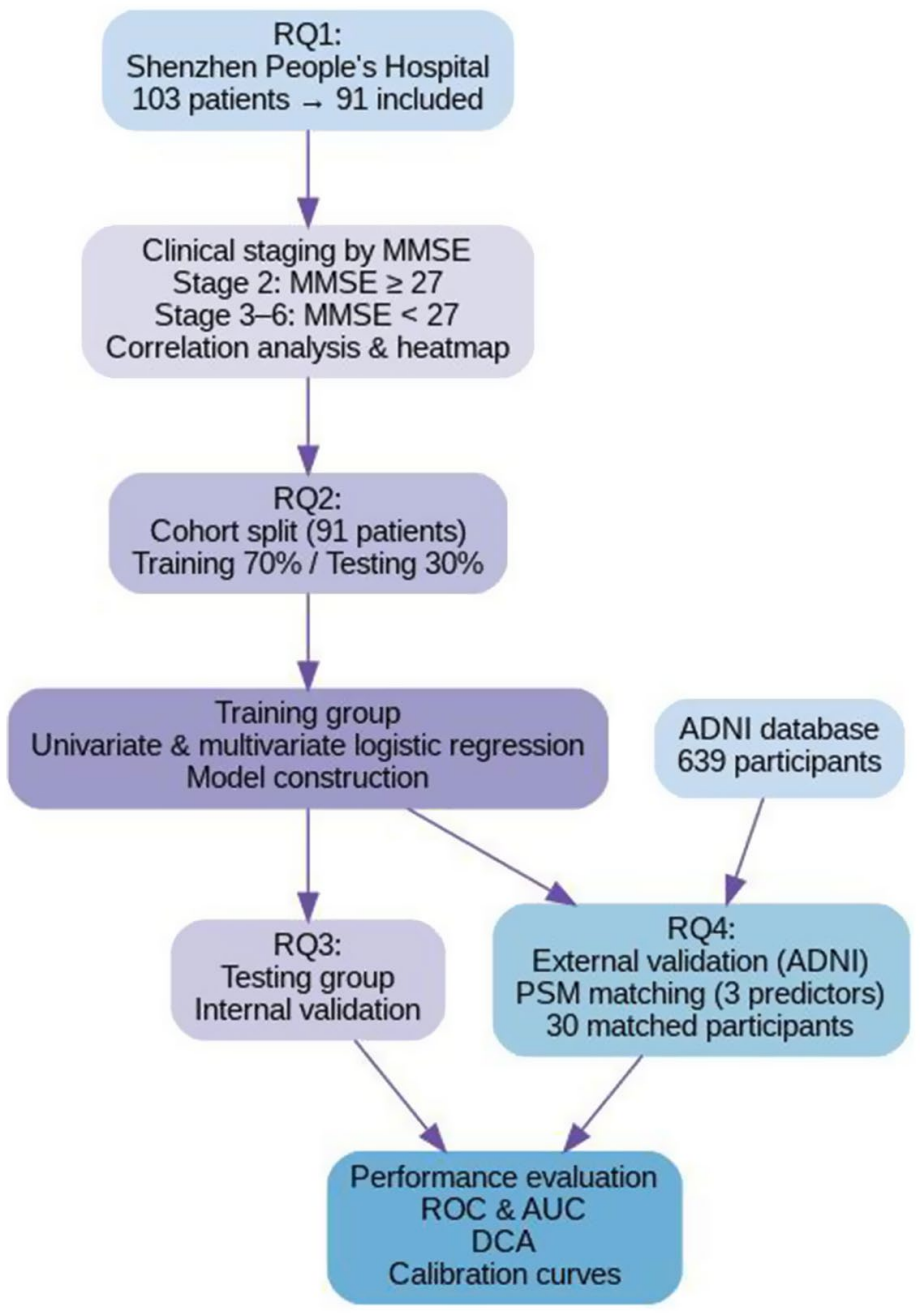
Study flowchart and research questions (RQs). RQ1 addressed participant selection and clinical staging: 103 patients from Shenzhen People’s Hospital were initially screened, with 91 meeting inclusion criteria after exclusions. Participants were classified as Clinical Stage 1–2 (MMSE ≥27; FAQ ≤5) or Clinical Stages 3–6 (MMSE ≤26; FAQ >5), followed by correlation analysis. RQ2 focused on model development: the cohort was randomly divided into training (70%) and testing (30%) sets, with logistic regression analyses performed in the training set to identify predictors for model construction. RQ3 involved internal validation using the testing set to assess model performance. RQ4 concerned external validation: 639 individuals from the ADNI database were initially considered, with 30 matched to the training set via propensity score matching (PSM) based on key predictors. Model performance was evaluated across training, testing, and external validation cohorts using ROC analysis (AUC), decision curve analysis (DCA), and calibration plots.

In accordance with the 2024 NIA-AA clinical staging framework (Jack et al., 2024), participants were categorized into two distinct groups: Clinical Stages 1–2 (*n* = 16), defined by subjective cognitive decline alongside preserved objective cognitive function (MMSE ≥ 27) and an FAQ score ≤5. Clinical Stages 3–6 (*n* = 75), encompassing individuals with objectively confirmed cognitive impairment spanning mild deficits to severe dementia (MMSE ≤ 26), accompanied by an FAQ score >5. The dataset was randomly divided into a training set (*n* = 63) and a testing set (*n* = 28) at a 7:3 ratio for model development and internal validation, respectively. The overall study design is schematically presented in Figure 1.

### 2.2 Data collection

Demographic, clinical, and laboratory parameters were systematically extracted from electronic medical records. Collected data included age, gender, height, weight, body mass index (BMI), comorbidities (hypertension and hyperlipidemia), systolic and diastolic blood pressure at admission, MMSE scores, CSF biomarkers (pTau181 and Aβ42), and peripheral blood biomarkers such as white blood cell count (WBC), platelet count (PLT), high-density lipoprotein cholesterol (HDL-C), and low-density lipoprotein cholesterol (LDL-C).

Based on established evidence, several derived hematological indices were also calculated and included: lymphocyte-to-monocyte ratio (LMR), platelet-to-lymphocyte ratio (PLR), neutrophil-to-lymphocyte ratio (NLR), prognostic nutritional index (PNI), albumin-to-globulin (A/G) ratio, triglyceride and glucose index (TyG), atherogenic index of plasma (AIP), systemic inflammation response index (SIRI), and systemic immune-inflammation index (SII). These indices have been implicated in nutritional status, metabolic regulation, and immune-inflammatory responses relevant to disease pathophysiology, with several markers previously associated with cognitive impairment (Dobiásová and Frohlich, 2001; Koyama et al., 2016; Cai et al., 2017; Maeda et al., 2019; Hong et al., 2021; Zhou et al., 2021; Dziedzic et al., 2022; Li et al., 2023; Gu et al., 2024; Inoue and Janini Gomes, 2024; Wang et al., 2024; Yang et al., 2024). Detailed formulas for calculating these derived indices are provided in the Supplementary Table S1.

### 2.3 Model development and evaluation

Prior to statistical analysis, the initial dataset was randomly divided into training and testing sets at a 7:3 ratio. The training set was used for model development, including parameter calibration, variable selection, and algorithmic optimization. The testing set was reserved for internal validation to assess model performance without any parameter adjustments. This approach allowed for evaluation of potential overfitting or underfitting and guided decisions regarding model retraining or alternative methodological approaches.

In the training set, both continuous and binary variables were subjected to univariate logistic regression analysis. The method for dichotomizing continuous variables is detailed in the subsequent section (Douglass, 2020; Demšar and Zupan, 2021). Variables demonstrating statistical significance (*p* < 0.1) were included in multivariate logistic regression analysis. Predictors retained in the final model were those maintaining significance (*p* < 0.1) in both univariate and multivariate analyses. Following this stepwise selection process, the identified predictors were incorporated into a multivariate logistic regression model.

A nomogram was developed from this final model to estimate the probability of AD progression to Clinical Stages 3–6. Model performance was evaluated in both the training and testing sets using discrimination and calibration metrics. Calibration curves were generated to compare the nomogram-predicted probabilities with observed outcomes. Discrimination, defined as the model’s ability to differentiate between Clinical Stage 1–2 and Stages 3–6, was assessed using the area under the receiver operating characteristic curve (AUC), with corresponding 95% confidence intervals calculated for both datasets.

The performance of the integrated model was compared against a CSF pTau181-only model. Additional performance metrics—including sensitivity, positive predictive value (PPV), and negative predictive value (NPV)—were calculated using the optimal probability threshold determined by maximizing the Youden index (J = sensitivity + specificity − 1) in the training set.

### 2.4 External validation using the ADNI cohort

For external validation of the predictive model, data were obtained from the Alzheimer’s Disease Neuroimaging Initiative (ADNI) database.^1^ ADNI was launched in 2003 as a public-private partnership under the leadership of Principal Investigator Michael W. Weiner, MD. The primary objective of ADNI is to determine whether serial magnetic resonance imaging (MRI), positron emission tomography (PET), other biological markers, and clinical assessments can effectively track the progression of MCI and early AD.

From the ADNI-1, ADNI-GO, ADNI-2, ADNI-3, and ADNI-4 studies, we initially identified 639 participants with available MMSE scores and serum/CSF biomarker data. To ensure comparability with our internal training set on key predictors, propensity score matching (PSM) was performed based on the variables selected in the final predictive model. This rigorous matching process resulted in a well-balanced external validation cohort. Clinical assessments, including MMSE scores, were conducted, and relevant biomarker analyses were performed at the Biomarker Research Laboratory, University of Pennsylvania, USA.

It should be noted that CSF measurements in the ADNI cohort were conducted using the Luminex xMAP platform (INNO-BIA AlzBio3 kit), whereas those in our internal cohort were performed with the INNOTEST® ELISA platform. Although these assays are known to generate differing absolute values, they have been demonstrated to be highly correlated and comparable in their ability to classify AD pathology (Irwin et al., 2012).

### 2.5 Statistical analysis

Descriptive statistics were first performed on the original dataset. The normality of all continuous variables was assessed using the Shapiro–Wilk test, and the homogeneity of variance was examined with Levene’s test. Based on these test results, variables were summarized and compared as follows: normally distributed continuous variables were presented as mean ± standard deviation and compared using the independent samples t-test; non-normally distributed continuous variables were expressed as median (first quartile, third quartile) [M (Q₁, Q_3_)] and analyzed with the Mann–Whitney U test; categorical variables were summarized as frequency and percentage [*n* (%)]. A significance level of *p* < 0.1 was applied for all baseline comparisons. Relationships between variables were examined using Spearman’s correlation analysis, with results visualized in a heatmap.

Univariate logistic regression was conducted for continuous variables, and receiver operating characteristic (ROC) curves were generated. Optimal cutoff values for the PNI index, PLR, and A/G ratio were determined by maximizing the Youden index (J = sensitivity + specificity − 1). Classification thresholds for other continuous variables were established based on previously published studies (Han et al., 2021; Gao et al., 2022; Mehta et al., 2023; Dowling et al., 2024).

## 3 Results

### 3.1 Baseline characteristics and univariate analysis

**Figure 2:**
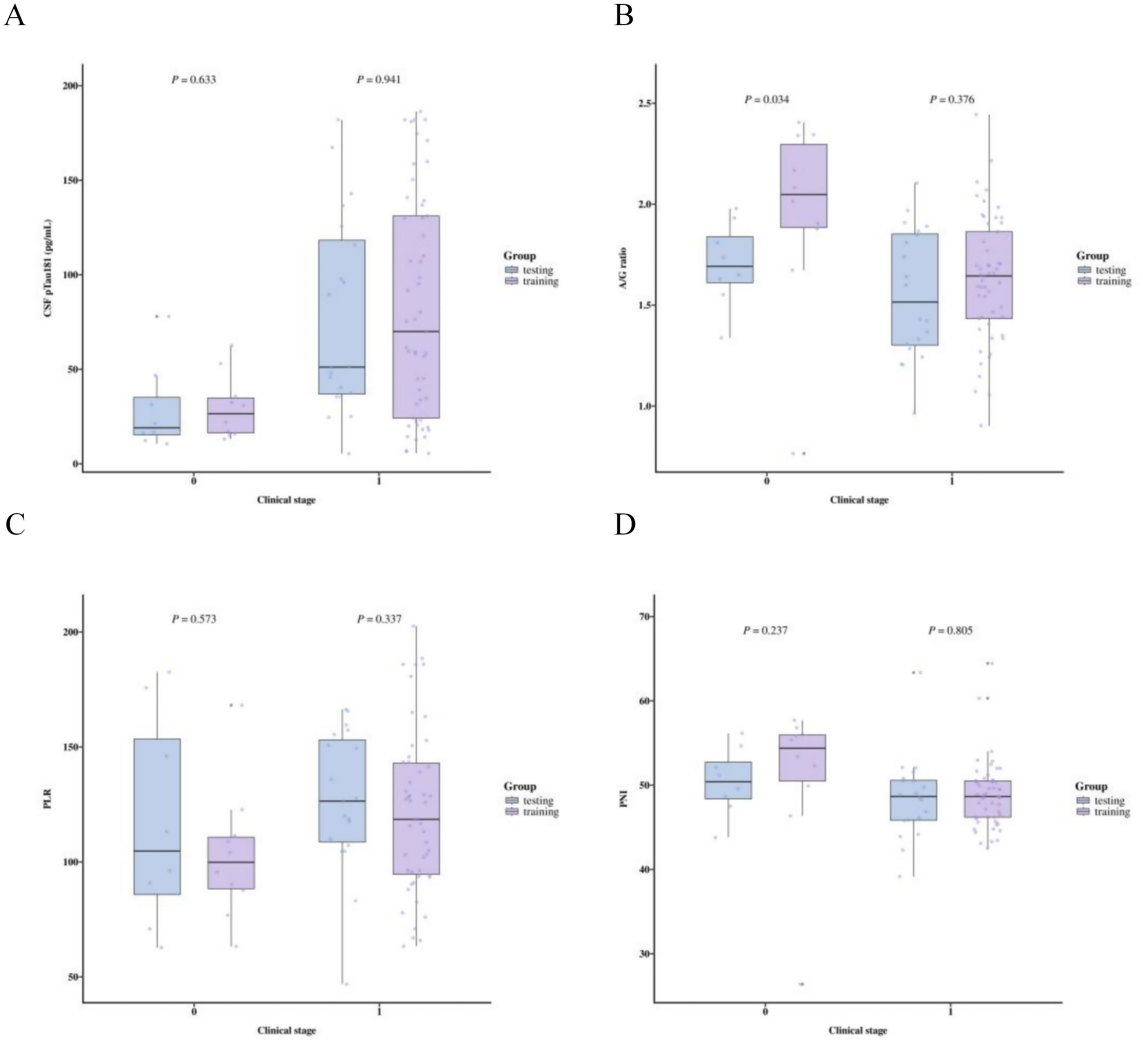
Comparison of variable distributions across clinical stages and between datasets. Box plots display the comparative levels of **(A)** CSF pTau181, **(B)** A/G ratio, **(C)** platelet-to-lymphocyte ratio (PLR), and **(D)** prognostic nutritional index (PNI) between clinical stage 1–2 (coded as 0) and clinical stages 3–6 (coded as 1). Group comparisons were performed using *t*-test for A/G ratio and Mann–Whitney U test for all other indicators. Statistically significant *p*-values are annotated.

**Figure 3:**
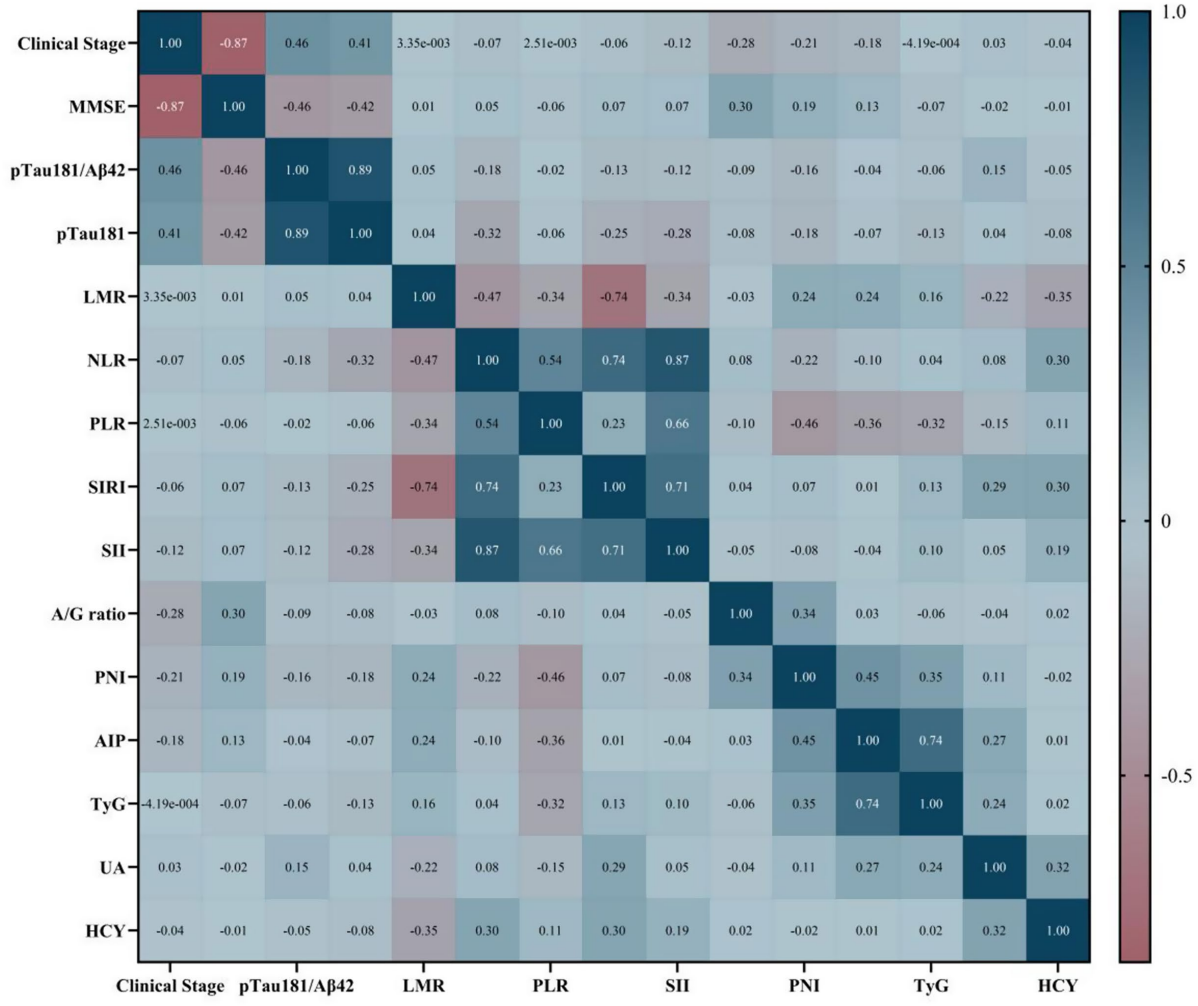
Spearman correlation heatmap of clinical stage and different variables. Spearman correlation heatmap depicting relationships between clinical stages, biomarkers, and metabolic indices, including MMSE scores, pTau181/A*β*42 ratio, inflammatory markers, and metabolic parameters. Color gradient represents correlation strength, with dark blue indicating strong positive correlations and dark red indicating strong negative correlations.

The baseline characteristics of the entire cohort, including both training and testing sets, are summarized in Table 1. In assessing the functional validity of the MMSE-based clinical staging, results demonstrated a significant gradient in functional impairment across the stratified groups, consistent with the underlying staging framework. Participants classified within Clinical Stages 3–6 exhibited markedly worse functional performance on the FAQ compared to those in Clinical Stages 1–2 (median [IQR]: 15 [12–21] vs. 1 [0–3]; Mann–Whitney U = 1194.5, *p* < 0.001). Comparative distributions of biomarker profiles between Clinical Stage 1–2 and Clinical Stages 3–6 are illustrated in Figure 2, while the results of the Spearman correlation analysis are summarized in Figure 3. Most demographic variables, clinical scores (e.g., MMSE), comorbidities, and biomarker profiles showed no significant differences between the training and testing sets (*p* > 0.1), supporting their comparability for subsequent model development. A statistically significant difference was observed in the arterial sclerosis index (AIP, *p* = 0.044), which was therefore excluded from further model development. Optimal cutoff values for the prognostic nutritional index (PNI), platelet-to-lymphocyte ratio (PLR), and albumin-to-globulin (A/G) ratio were determined by maximizing the Youden index. Based on these thresholds, participants were categorized as follows: PLR ≥ 113.22 was classified as the high-PLR group, PNI ≤ 52.05% as the low-PNI group, and A/G ratio ≤1.62 as the low A/G group. Univariate logistic regression analysis identified serum biomarkers significantly associated with advanced clinical stages (Stages 3–6 versus Stages 1–2; *p* < 0.1). Variables meeting this criterion were entered into an initial multivariable logistic regression model. To ensure model stability and interpretability, variable selection was performed in a stepwise manner: first, the pTau181/Aβ42 ratio was excluded due to high collinearity with CSF pTau181, which attenuated the statistical significance of both terms when included simultaneously. Subsequently, both the A/G ratio ≤1.62 and the PNI ≤ 52.05% lost statistical significance after adjustment and were therefore omitted. This selection process yielded three predictors in the final model: two continuous variables (CSF pTau181 and A/G ratio) and one dichotomized variable (high PLR group). Detailed results of the univariate and multivariable analyses are presented in Table 2.

**Table 1:** Baseline characteristics of the study participants.

Characteristics	Total (*n* = 91)	Training (*n* = 63)	Testing (*n* = 28)	*p*
Sex (male), %	52 (57.14)	20 (71.43)	32 (50.79)	0.066
Age at AD diagnosis, years	63.69 ± 8.11	63.50 ± 9.20	63.78 ± 7.66	0.881
Height, m	1.63 ± 0.07	1.65 ± 0.08	1.63 ± 0.07	0.130
Weight, Kg	65.00 (52.50, 70.15)	65.50 (51.00, 71.62)	64.30 (54.00, 69.65)	0.813
BMI, kg/m^2^	23.49 (20.53, 25.10)	23.24 (19.87, 25.59)	23.49 (20.85, 24.66)	0.708
SBP, mmHg	129.00 (118.50, 148.00)	132.00 (121.50, 151.75)	127.00 (117.50, 148.00)	0.351
DBP, mmHg	81.00 (74.50, 90.00)	82.50 (75.50, 90.00)	81.00 (74.50, 89.00)	0.670
MMSE total score	20.00 (13.00, 26.00)	22.50 (14.75, 26.00)	20.00 (11.50, 25.50)	0.399
FAQ total score	13.835 ± 0.8.761	14.317 ± 9.128	12.75 ± 7.924	0.434
Number of Clinical Stages 3–6, %	75 (82.42)	22 (78.57)	53 (84.13)	0.731
Number of HTN, %	44 (48.35)	14 (50.00)	30 (47.62)	0.834
Number of HLD, %	12 (13.19)	2 (7.14)	10 (15.87)	0.423
CSF pTau181, Pg/ml	51.51 (22.88, 112.91)	46.30 (24.96, 96.41)	58.39 (22.24, 125.22)	0.547
Aβ42, Pg/ml	428.56 (315.35, 690.57)	419.67 (315.35, 662.77)	493.02 (321.69, 858.86)	0.483
pTau181/Aβ42	0.12 (0.04, 0.26)	0.12 (0.05, 0.27)	0.10 (0.03, 0.19)	0.378
LMR	3.77 (3.04, 5.27)	3.64 (3.14, 4.31)	3.83 (2.99, 5.75)	0.216
NLR	1.87 (1.45, 2.70)	1.94 (1.44, 2.79)	1.87 (1.47, 2.53)	0.767
PLR	118.62 (95.61, 150.04)	123.24 (104.65, 155.95)	116.67 (93.97, 143.51)	0.431
PNI, %	49.00 (46.20, 51.38)	48.95 (46.04, 51.29)	49.00 (46.23, 51.42)	0.833
A/G ratio	1.65 ± 0.34	1.60 ± 0.30	1.68 ± 0.36	0.300
TyG	8.50 (8.14, 8.93)	8.62 (8.16, 9.11)	8.44 (8.14, 8.82)	0.513
AIP	2.47 (2.00, 3.51)	2.40 (1.79, 4.09)	2.51 (2.12, 3.49)	0.979
Hcy, μmol/L	13.00 (10.90, 16.15)	13.40 (11.38, 14.88)	12.80 (10.15, 16.30)	0.683
UA, μmol/L	328.90 ± 86.16	332.08 ± 101.08	327.48 ± 79.49	0.816
Number of PNI ≤ 52.05%, %	72 (79.12)	22 (78.57)	50 (79.37)	0.931
Number of AIP ≥ 4, %	14 (15.38)	8 (28.57)	6 (9.52)	0.044
Number of A/G ratio ≤1.62, %	39 (42.86)	13 (46.43)	26 (41.27)	0.646
Number of LMR ≤ 3.5, %	36 (39.56)	25 (39.68)	11 (39.29)	0.971
Number of NLR ≥ 3, %	14 (15.38)	11 (17.46)	3 (10.71)	0.611
Number of PLR ≥ 113.22, %	51 (56.04)	34 (53.97)	17 (60.71)	0.550
SIRI	0.85 (0.57, 1.33)	0.91 (0.65, 1.55)	0.80 (0.56, 1.19)	0.308
SII	381.85 (275.69, 574.53)	387.09 (309.17, 539.53)	380.68 (258.90, 596.17)	0.627

**Table 2:** The results of univariate and multivariate logistic regression.

Variables	Univariate					Multivariate				
	*β*	S.E	*Z*	*p*	OR (95%CI)	*β*	S.E	*Z*	*p*	OR (95%CI)
pTau181 (Pg/ml)	0.03	0.01	2.02	0.044	1.03 (1.01–1.06)	0.04	0.02	1.98	0.048	1.04 (1.01–1.09)
pTau181/Aβ42	12.14	6.44	1.88	0.060	186530.79 (0.61–56831166178.20)					
LMR	−0.14	0.24	−0.61	0.545	0.87 (0.54–1.38)					
NLR	−0.12	0.44	−0.28	0.780	0.88 (0.37–2.11)					
PLR	0.02	0.02	1.57	0.116	1.03 (0.99–1.06)					
BMI (kg/m^2^)	−0.00	0.10	−0.03	0.975	1.00 (0.82–1.22)					
SIRI	−0.02	0.87	−0.02	0.981	0.98 (0.18–5.39)					
SII	0.00	0.00	0.57	0.570	1.00 (1.00–1.01)					
A/G ratio	−2.43	1.25	−1.94	0.053	0.09 (0.01–1.03)	−2.46	1.44	−1.71	0.088	0.09 (0.01–1.44)
PNI (%)	−0.03	0.07	−0.35	0.729	0.97 (0.84–1.13)					
TyG	−0.08	0.71	−0.12	0.904	0.92 (0.23–3.67)					
UA (μmol/L)	−0.00	0.00	−0.48	0.631	1.00 (0.99–1.01)					
HCY (μmol/L)	−0.12	0.08	−1.43	0.152	0.89 (0.75–1.05)					
LMR ≤ 3.5	0.41	0.89	0.46	0.649	1.50 (0.26–8.58)					
NLR ≥ 3	−0.13	1.19	−0.11	0.911	0.87 (0.08–9.07)					
PLR ≥ 113.22	1.95	1.12	1.74	0.082	7.00 (0.78–62.84)	2.59	1.27	2.04	0.041	13.37 (1.11–161.49)
A/G ratio ≤1.62	1.95	1.12	1.74	0.082	7.00 (0.78–62.84)					
PNI ≤ 52.05%	2.71	0.93	2.91	0.004	15.00 (2.42–93.01)					

### 3.2 Predictive model and performance

**Figure 4:**
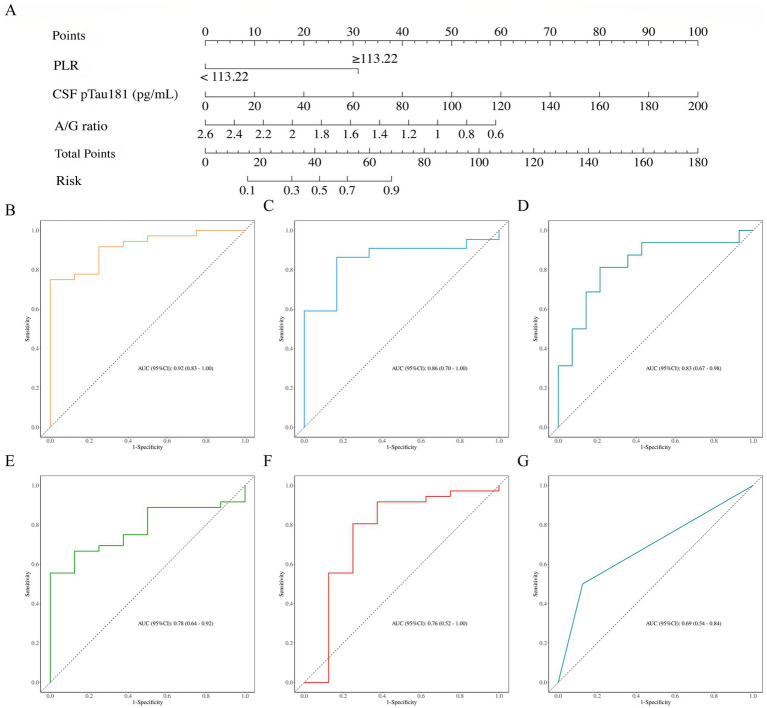
Performance evaluation of the integrated model and its individual components. **(A)** Nomogram of the final integrated model for discriminating between AD clinical stages 3–6 and stage 2. Receiver operating characteristic (ROC) curves demonstrate the discriminatory performance of **(B)** the integrated model in the training set (AUC = 0.92, 95% CI: 0.83–1.00), **(C)** the integrated model in the testing set (AUC = 0.86, 95% CI: 0.70–1.00), and **(D)** the external validation set (AUC = 0.83, 95% CI: 0.67–0.98). Comparative ROC analyses show the performance of **(E)** CSF pTau181 alone (AUC = 0.78, 95% CI: 0.64–0.92), **(F)** the albumin-to-globulin (A/G) ratio (AUC = 0.76, 95% CI: 0.52–1.00), and **(G)** the platelet-to-lymphocyte ratio (PLR ≥ 113.22; AUC = 0.69, 95% CI: 0.54–0.84).

The integrated model, developed from multivariate logistic regression in the training set, demonstrated excellent discrimination for identifying Clinical Stages 3–6, with an area under the receiver operating characteristic curve (AUC) of 0.92 (95% CI: 0.83–1.00; Figure 4B). The model maintained robust performance in the independent testing set, achieving an AUC of 0.86 (95% CI: 0.70–1.00; Figure 4C). Notably, the combined model showed significantly improved discrimination compared against a CSF pTau181-only model (AUC = 0.78, 95% CI: 0.64–0.92; Figures 4E-G). Additional performance metrics of the final model on the testing set are listed in Table 3.

**Table 3:** Performance of the integrated predictive model in the testing set.

AUC (95%CI)	Accuracy (95%CI)	Sensitivity (95%CI)	Specificity (95%CI)	PPV (95%CI)	NPV (95%CI)	Cutoff
0.86 (0.70–1.00)	0.86 (0.67–0.96)	0.83 (0.54–1.00)	0.86 (0.72–1.00)	0.62 (0.29–0.96)	0.95 (0.85–1.00)	0.646

**Figure 5:**
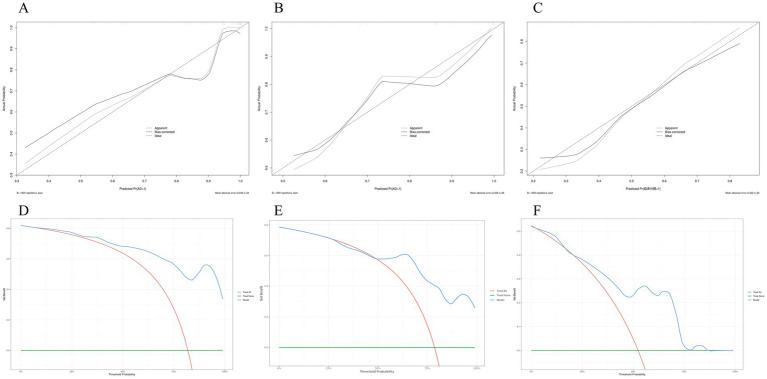
Calibration and clinical utility of the integrated predictive model. Calibration curves for the integrated model in the **(A)** training set, **(B)** testing set, and **(C)** external validation set. The dashed 45-degree line represents ideal calibration, while the solid bias-corrected line indicates model performance after overfitting adjustment. Closer alignment with the diagonal indicates better agreement between predicted probabilities and observed outcomes. Decision curve analysis (DCA) evaluating the clinical utility of the integrated model in the **(D)** training set, **(E)** testing set, and **(F)** external validation set. The DCA curves demonstrate the net benefit of the model across different threshold probabilities.

A nomogram was constructed based on the final multivariate regression model to visually estimate the probability of an AD patient belonging to Clinical Stages 3–6 (Figure 4A). Calibration performance in the testing cohort showed strong agreement between predicted probabilities and observed outcomes. The bias-corrected curve, derived from 1,000 bootstrap resamples, closely followed the ideal calibration line, with a mean absolute error (MAE) of 0.039, indicating minimal deviation between predictions and observations (Figures 5A,B). Decision curve analysis (DCA) revealed that the model provided higher net benefit than the “treat all” strategy across a wide range of threshold probabilities, although within a narrow interval (approximately 25–50%), the net benefit was marginally lower than that of the “treat all” approach (Figures 5D,E).

### 3.3 External validation with propensity score matching

**Figure 6:**
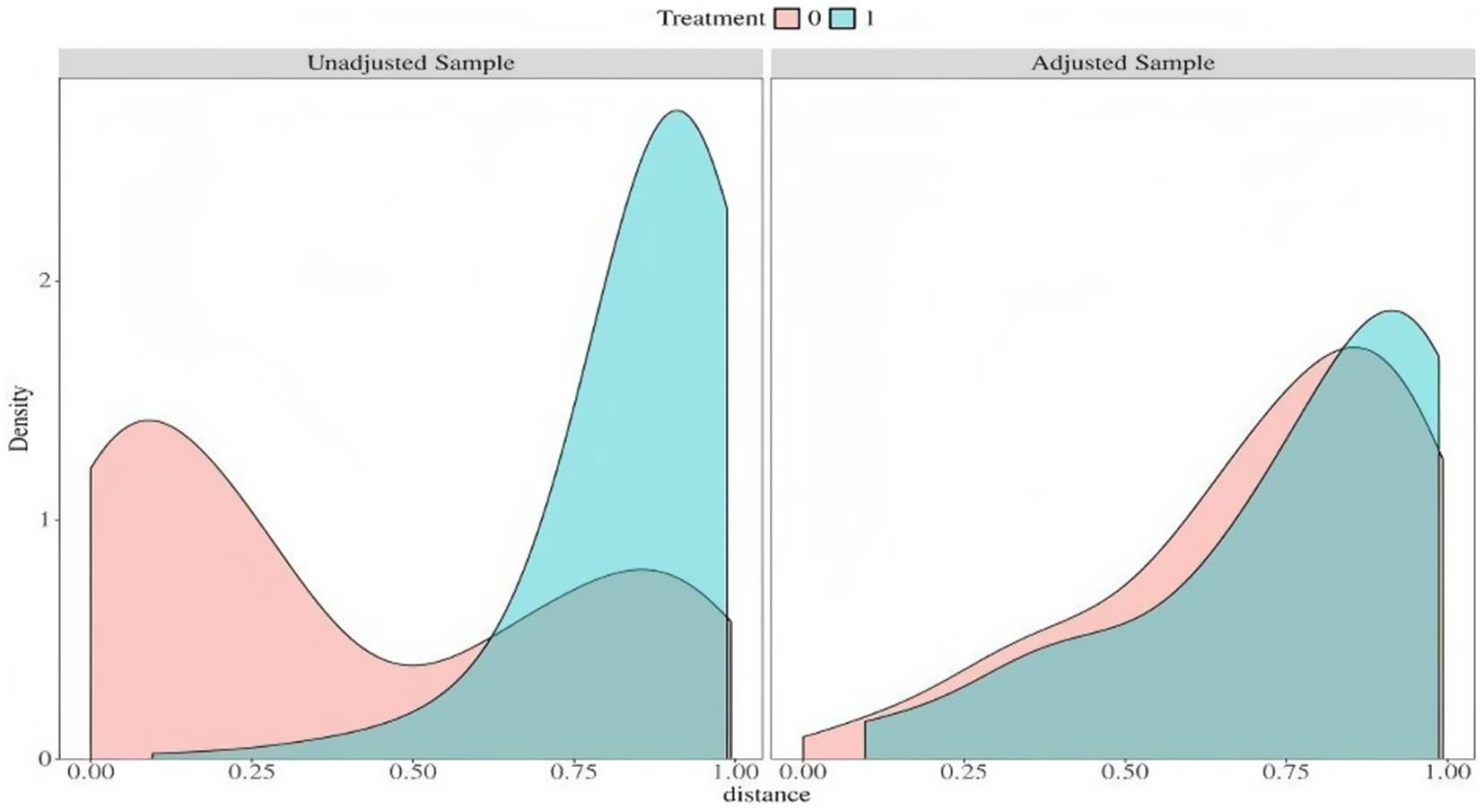
Distribution balance for distance after propensity score matching. Density plots illustrating the distribution of propensity scores between the internal training set (labeled as 1) and the ADNI external validation cohort (labeled as 0). The left panel shows the initial imbalance in the unmatched sample, while the right panel demonstrates substantially improved balance achieved after propensity score matching.

External validation was conducted using data from the ADNI cohort, which initially included 639 individuals. Propensity score matching (PSM) was applied to align the ADNI samples with the training set based on three key predictors in the final model: CSF pTau181, A/G ratio, and high PLR status (PLR ≥ 113.22). After matching, 30 individuals from the ADNI cohort were successfully paired with training set participants, achieving balanced distributions as indicated by minimal standardized mean differences (SMDs; Figure 6; Table 4).

**Table 4:** Propensity score matching results for the external validation set.

Variable	Total (*n* = 60)	training (*n* = 30)	validation (*n* = 30)	*p*	SMD
A/G ratio, Mean ± SD	1.49 ± 0.35	1.52 ± 0.32	1.46 ± 0.37	0.490	−0.171
CSF pTau181, M (Q_1_, Q_3_)	22.88 (16.25, 38.95)	22.49 (15.96, 39.08)	25.35 (17.12, 38.54)	0.963	−0.244
PLR ≥ 113.22, *n* (%)	16 (27.59)	10 (34.48)	6 (20.69)	0.240	−0.341

In the external validation set, the model exhibited robust discriminatory power with an AUC of 0.83 (95% CI: 0.67–0.98) despite the inter-platform assay differences (Figure 4D). Moreover, its clinical utility and calibration were consistently affirmed by decision curve and calibration curve analyses within the same cohort (Figures 5C,F).

## 4 Discussion

In this study, we developed and validated a predictive model for AD clinical progression by integrating CSF pTau181 with serum biomarkers—the albumin-to-globulin (A/G) ratio and the platelet-to-lymphocyte ratio (PLR). The model demonstrated robust discrimination and good calibration across both internal and external validation cohorts, highlighting the contributions of nutritional status and systemic inflammation to the AD clinical continuum.

### 4.1 Nutritional status and the A/G ratio

Our findings indicate that a higher A/G ratio is associated with a reduced risk of AD progression, suggesting a potential protective role. The A/G ratio reflects the balance between protein nutrition, represented by albumin, and immune activity, represented by globulin. An elevated A/G ratio typically signifies adequate nutritional reserves and antioxidant capacity, whereas a lower ratio may indicate chronic inflammation or malnutrition. This balance may be modulated by the brain-gut axis, through which gut health influences systemic inflammation and nutrient absorption—key determinants of albumin and globulin levels (Zhou et al., 2022).

Previous epidemiological studies have consistently linked a lower A/G ratio to an increased risk of cognitive impairment (Koyama et al., 2016; Maeda et al., 2019). Recent analyses of NHANES data further support a non-linear relationship between the A/G ratio and cognitive function in older adults (Yang et al., 2024). Our results support and extend these observations, reinforcing the notion that nutritional status serves as a modifiable protective factor against AD progression.

Mechanistically, albumin not only functions as a transport protein but also exhibits antioxidant and anti-inflammatory properties, enabling it to scavenge free radicals and bind amyloid proteins. The promising results from the AMBAR clinical trial, which demonstrated benefits of albumin-based plasma exchange in AD patients, provide direct human evidence supporting the neuroprotective and anti-Aβ roles of albumin, potentially mediated through stabilization of the blood–brain barrier (Boada et al., 2020). Furthermore, malnutrition can impair neuronal resilience by limiting substrates essential for synaptic maintenance. Experimental studies have shown that protein-energy deficiency downregulates the expression of key synaptic proteins (e.g., BDNF, GAP-43) and induces detrimental hippocampal ultrastructural changes, deficits that can be partially reversed by dietary supplementation (Prosser-Loose et al., 2010; Sato et al., 2021).

Despite the strong biological plausibility, some caution is warranted when extrapolating these findings beyond the study population. The primary cohort was recruited from a single hospital in China, and systemic inflammatory and nutritional biomarkers may vary across populations owing to differences in ethnicity, dietary patterns, and healthcare context. Although external validation was conducted using the ADNI cohort, this analysis was retrospective and based on a limited subset of the overall ADNI population, which may constrain direct translation to real-world clinical settings. Thus, while the observed biological associations appear robust, the predictive performance of the model requires further evaluation in diverse, prospective, multicenter cohorts.

Clinically, these findings underscore the importance of nutritional interventions in AD management. Substantial evidence has demonstrated that dietary patterns such as the Mediterranean diet—rich in vegetables, fruits, whole grains, and fish—significantly reduce the risk of cognitive decline and AD (Scarmeas et al., 2018). Our study provides a mechanistic rationale for such interventions, suggesting that maintaining an optimal A/G ratio through adequate nutrition may represent a viable strategy to slow disease progression.

### 4.2 Systemic inflammation and the PLR

Conversely, we found that an elevated PLR was associated with a higher risk of AD progression. The PLR reflects the balance between pro-inflammatory activity (represented by platelets) and immune regulation (represented by lymphocytes). A high PLR suggests a state of enhanced systemic inflammation coupled with compromised immune protection.

Systemic inflammation is postulated to exacerbate AD pathology through peripheral-central immune crosstalk. This process may be further amplified by the disruption of brain energy metabolism, as systemic inflammation can impair cerebral glucose utilization and mitochondrial function, thereby aggravating neuronal vulnerability and pathological progression (Yu et al., 2022). Potential mechanisms include: (1) systemic inflammation activating inflammatory pathways (e.g., the GSDMD signaling) in cerebral endothelial cells, leading to blood–brain barrier damage (Wei et al., 2024); (2) peripheral inflammatory mediators infiltrating the central nervous system, activating microglia and triggering neuroinflammation and tau hyperphosphorylation (Heneka et al., 2015; Leng and Edison, 2021); (3) disruption of immune homeostasis impairing intrinsic neuroprotective mechanisms. Our results align with previous studies linking elevated peripheral inflammatory markers, including PLR and NLR, to cognitive decline, brain atrophy, and Aβ deposition (Han et al., 2021; Li et al., 2023; Zheng et al., 2025). Therefore, in addition to nutritional support, anti-inflammatory strategies—such as dietary modifications rich in polyunsaturated fatty acids and polyphenols, and early management of comorbid inflammatory conditions—should be emphasized in AD care.

### 4.3 Model integration and clinical implications

The integration of CSF pTau181, a core biomarker of AD neurofibrillary pathology, with peripheral indicators of nutrition (A/G ratio) and inflammation (PLR), provides a more holistic view of the disease. This combined model suggests that AD clinical progression is driven not only by central tau pathology but also by interplay with systemic physiological states. This multi-system perspective aligns with emerging research and could enhance risk stratification in clinical practice, potentially identifying patients who might benefit from integrated management strategies targeting both central pathology and peripheral modifiers.

### 4.4 Limitations

Several limitations of this study should be acknowledged. First, the relatively small sample size of our primary cohort increases the risk of model overfitting, and the wide confidence intervals observed in the testing set warrant cautious interpretation. In addition, although external validation was conducted using the ADNI dataset, the matched validation cohort constitutes only a small subset of the overall ADNI population. A further methodological consideration pertains to the absence of a formal prospective sample size calculation. While all eligible participants from available retrospective datasets were included, the resulting cohort size remains below the thresholds typically recommended in contemporary guidelines for fully optimized predictive modeling. Given the limited dataset, the dichotomization of selected continuous variables represents a pragmatic trade-off to enable construction of a bedside-friendly nomogram, while more complex machine-learning approaches are not suitable for small-sample modeling. Consequently, the current model should be interpreted as a preliminary proof of concept. External validation in larger, independent cohorts will be essential to confirm the generalizability of the findings (Riley et al., 2020; Pate et al., 2023; Efthimiou et al., 2024). Second, the primary aim of the nomogram is to facilitate risk stratification, not to direct individualized treatment. In line with this function, we observed a decline in net benefit across intermediate predicted probability ranges (25–50%). Thus, the model may offer limited clinical utility for patients within this interval, thereby indicating its greater applicability at the lower and upper extremes of the risk spectrum. Third, although Aβ pathology is a cornerstone of AD, Aβ markers were not retained in our final multivariate model, likely due to statistical reasons rather than biological irrelevance (Janelidze et al., 2016). Previous studies have underscored the high diagnostic value of the pTau181/Aβ42 ratio in both CSF and serum (Ortner et al., 2022; Lehmann et al., 2025), and our Spearman correlation analysis also confirmed a significant association between this ratio and AD clinical stages. Nonetheless, it did not retain statistical significance in the final multivariate logistic regression, and therefore was excluded from model construction. Certain factors, such as APOE genotype, vascular comorbidities, and neuroimaging biomarkers, were intentionally omitted from the model. This strategic omission represents a pragmatic design choice to ensure clinical feasibility and scalability by relying on more readily obtainable CSF and routine blood-based measures.

Fourth, Phosphorylated tau at threonine 181 (pTau181) is a core CSF biomarker of AD–related neurofibrillary pathology. Its positive association with disease presence and severity has been consistently demonstrated across diverse populations and analytical platforms, underscoring its biological robustness and generalizability (Janelidze et al., 2020).

The Luminex xMAP platform was among the first high-throughput immunoassay systems adopted for quantifying CSF tau and pTau181. Its clinical relevance to AD severity and neuropathological burden was established in large multicenter cohorts, including the Alzheimer’s Disease Neuroimaging Initiative (ADNI), during the mid- to late-2000s (Olsson et al., 2005; Shaw et al., 2009). Subsequently, enzyme-linked immunosorbent assay (ELISA)–based methods—valued for their technical simplicity, commercial availability, and high analytical stability—have gained widespread use in both research and clinical laboratory practice. These assays have likewise demonstrated strong correlations between CSF pTau181 levels and AD severity (Blennow et al., 2010; Janelidze et al., 2020).

Notably, methodological comparisons have shown that although ELISA- and xMAP-based assays yield systematically different absolute concentrations, measurements from the two platforms are highly correlated and exhibit comparable diagnostic performance for AD pathology (Irwin et al., 2012; Lleó et al., 2018). These observations indicate that both analytical approaches reliably capture the same underlying biological process—namely, the progressive increase in pTau181 along the AD continuum.

In the present study, despite differences in the CSF pTau181 assay platform between the internal and external (ADNI) cohorts, the predictive model retained good discriminative performance in the external validation set (area under the curve [AUC] = 0.83, 95% confidence interval [CI]: 0.67–0.98; Figure 4D). Consistent calibration and favorable decision-curve analyses further supported the model’s robustness in this independent population (Figures 5C,F). Accordingly, the primary objective of the external validation was not to compare absolute biomarker values across platforms, but to evaluate whether the predictive relationship identified by the model—which integrates CSF pTau181 with the serum albumin-to-globulin ratio and platelet-to-lymphocyte ratio—could be generalized to an independently ascertained cohort. The maintained performance in the ADNI cohort suggests that this integrative biological signal remains informative even when the central biomarker component is quantified using a different, yet validated, analytical platform. It should be noted, however, that inter-platform differences may influence absolute risk estimation; therefore, platform-specific recalibration would be necessary before clinical translation.

## 5 Conclusion

Despite these limitations, our integrated model marks meaningful advance toward personalized and accessible AD prognosis. It establishes a practical and accessible framework that assists clinicians in identifying AD patients who have progressed to Clinical Stages 3–6, thereby facilitating more accurate prognostic evaluation and tailored treatment planning. It should be noted that the present model is designed to identify the transition to the stage of objective cognitive impairment. The separate and distinct question of predicting the rate or likelihood of progression from mild cognitive impairment (MCI) to dementia warrants future investigation through dedicated longitudinal study designs. Furthermore, our findings contribute to a deeper understanding of the role played by nutritional status and immune response in the pathophysiological progression of AD, providing a basis for future research on integrated, multi-dimensional approaches to AD care and intervention.

## Data Availability

The raw data supporting the conclusions of this article will be made available by the authors, without undue reservation.

## Footnotes

1
adni.loni.usc.edu

## Group members of Alzheimer’s Disease Neuroimaging Initiative

Data used in preparation of this article were obtained from the Alzheimer’s Disease Neuroimaging Initiative (ADNI) database (adni.loni.usc.edu). As such, the investigators within the ADNI contributed to the design and implementation of ADNI and/or provided data but did not participate in analysis or writing of this report. A complete listing of ADNI investigators can be found at: http://adni.loni.usc.edu/wp-content/uploads/how_to_apply/ADNI_Acknowledgement_List.pdf.
