# Supplementary material for: An integrated CSF-serum biomarker model for predicting clinical progression in Alzheimer’s disease
Source: Front Aging Neurosci. 2026 Jan 27;18:1728675. doi: 10.3389/fnagi.2026.1728675 (PMC12888026; doi:10.3389/fnagi.2026.1728675)
Supplement: Supplementary file 1 [file Table_1.DOCX]

Supplementary Material

# Supplementary Figures and Tables

## Supplementary Tables

**Supplementary Table 1.** **The calculation methods** **of the variables mentioned in the article.**

| Variable | Calculation methods |
| --- | --- |
|  |  |
| Albumin-to-globulin ratio (A/G) | = albumin / globulin |
| Prognostic Nutritional Index (PNI) | = serum albumin concentration (g/L) + 5 × total peripheral blood lymphocyte count (×10^9/L) |
| Lymphocyte-to-monocyte ratio (LMR) | = lymphocyte count / monocyte count |
| Neutrophil-to-lymphocyte ratio (NLR) | = neutrophil count / lymphocyte count |
| Platelet-to-lymphocyte ratio (PLR) | = platelet count / lymphocyte coun |
| Systemic immune-inflammation index (SII) | = neutrophil count × platelet count / lymphocyte count |
| Systemic inflammation response index (SIRI) | = neutrophil count × monocyte count / lymphocyte count |
| Atherogenic index of plasma (AIP) | = (total cholesterol – HDL-C) / HDL-CTyG |
| Triglyceride and glucose index (TyG) | = ln[fasting triglycerides (mg/dL) × fasting glucose (mg/dL)/2] |
